# Peripheral Blood Mononuclear Cells: A New Frontier in the Management of Patients with Diabetes and No-Option Critical Limb Ischaemia

**DOI:** 10.3390/jcm12196123

**Published:** 2023-09-22

**Authors:** Marco Meloni, Laura Giurato, Aikaterini Andreadi, Ermanno Bellizzi, Alfonso Bellia, Davide Lauro, Luigi Uccioli

**Affiliations:** 1 Department of Systems Medicine, University of Rome “Tor Vergata”, 00133 Rome, Italyermannobellizzi27@gmail.com (E.B.); bellia@med.uniroma2.it (A.B.); d.lauro@med.uniroma2.it (D.L.); 2 Division of Endocrinology and Diabetology, Department of Medical Sciences, Fondazione Policlinico “Tor Vergata”, 00133 Rome, Italy; 3 Division of Endocrinology and Diabetes, CTO Andrea Alesini Hospital, 00145 Rome, Italy; lauragiurato@yahoo.it (L.G.);; 4 Department of Biomedicine and Prevention, University of Rome Tor Vergata, 00133 Rome, Italy

**Keywords:** diabetes, diabetic foot, critical limb ischaemia, cell therapy

## Abstract

The current study aimed to evaluate the effectiveness of peripheral blood mononuclear cell (PB-MNC) therapy as adjuvant treatment for patients with diabetic foot ulcers (DFUs) and no-option critical limb ischaemia (NO-CLI). The study is a prospective, noncontrolled, observational study including patients with neuro-ischaemic DFUs and NO-CLI who had unsuccessful revascularization below the ankle (BTA) and persistence of foot ischaemia defined by TcPO2 values less than 30 mmHg. All patients received three cycles of PB-MNC therapy administered through a “*below-the-ankle approach*” in the affected foot along the wound-related artery according to the angiosome theory. The primary outcome measures were healing, major amputation, and survival after 1 year of follow-up. The secondary outcome measures were the evaluation of tissue perfusion by TcPO2 and foot pain defined by the numerical rating scale (NRS). Fifty-five patients were included. They were aged >70 years old and the majority were male and affected by type 2 diabetes with a long diabetes duration (>20 years); the majority of DFUs were infected and nearly 90% were assessed as gangrene. Overall, 69.1% of patients healed and survived, 3.6% healed and deceased, 10.9% did not heal and deceased, and 16.4% had a major amputation. At baseline and after PB-MNC therapy, the TcPO2 values were 17 ± 11 and 41 ± 12 mmHg, respectively (*p* < 0.0001), while the pain values (NRS) were 6.8 ± 1.7 vs. 2.8 ± 1.7, respectively (*p* < 0.0001). Any adverse event was recorded during the PB-MNC therapy. Adjuvant PB-MNC therapy seems to promote good outcomes in patients with NO-CLI and neuro-ischaemic DFUs.

## 1. Introduction

Recently, an increase in ischaemic diabetic foot ulcers (DFUs) has been documented, involving nearly 70% of diabetic patients who were referred to a specialized diabetic foot service for a new foot ulcer [1]. In addition, ischaemic DFUs are associated with worse outcomes in comparison to neuropathic DFUs in terms of healing, amputation, and survival [1,2].

In recent years, many improvements have been achieved in the management of ischaemic DFUs, mainly through a concomitant increase in lower limb revascularization, which allowed a reduction in the rate of major amputation [3]. Although lower limb revascularization is a common and successful procedure in dedicated settings, nowadays, revascularization is not feasible in 15–25% of patients due to the inability to overcome stenosis or an obstruction of leg/foot arteries [4]. Unsuccessful revascularization is closely related to major amputation [4] and this category of patients are defined as no-option critical limb ischaemia (NO-CLI).

Nearly 30% of NO-CLI patients report a 1-year major amputation and are characterized by several comorbidities (mainly dialysis) and a multisegmental disease, and more than 70% show an involvement of below-the-ankle (BTA) arteries [4]. BTA arterial disease is the most severe pattern of CLI, and it has been documented to be an independent predictor of healing and amputation [5,6]. In patients with ischaemic DFUs and BTA arterial disease who have failed foot revascularization, 9.1% reported healing and 36.3% reported a major amputation at 1-year follow-up [7].

In the last decade, autologous cell therapy (ACT), specifically peripheral blood mononuclear cell (PB-MNC) therapy, has been used in NO-CLI, with promising results reported in terms of healing, limb salvage, and survival [8,9]. Although the procedure seems safe and effective, PB-MNC therapy has been used in different settings, different subsets of patients, and without homogeneous criteria among clinical studies.

To the best of our knowledge, there are poor data in the literature about the use of cell therapy exclusively in very complex patients with BTA arterial disease.

Accordingly, the current study aimed to evaluate the effectiveness of PB-MNC therapy as a rescue treatment for patients with ischaemic DFUs and NO-CLI who had unsuccessful BTA revascularization.

## 2. Materials and Methods

The current study is a prospective observational noncontrolled study including consecutive patients affected by ischaemic/neuro-ischaemic DFUs and NO-CLI, enrolled between March 2019 and June 2022, who had unsuccessful lower limb revascularization.

All patients had been managed in the Department of Endocrinology, Diabetology and Metabolic disease, a tertiary-level diabetic foot service, at the Polyclinic of “Tor Vergata” University serving Rome, Italy.

The included patients were those with ischaemic/neuro-ischaemic DFUs belonging to stage 1C, 2C, 3C and 1D, 2D, 3D of the Texas University Classification [10] who had unsuccessful technical BTA revascularization, defined as the inability to overcome the stenosis and/or obstruction of diseased foot arteries. Accordingly, patients considered for this study were those with desert foot (an absence of any vessels BTA such as the pedal artery and plantar arteries) and/or those with a failed direct revascularization of “wound related artery” with persistent foot ischemia in the wound angiosome area defined by TcPO2 levels below 30 mmHg [11]. In addition, only patients with the patency of at least one artery below-the-knee (anterior tibial artery, peroneal artery, and posterior tibial artery) were included (Table 1).

The excluded patients were those with an indication for primary amputation due to the absence of anatomical suitability for surgical foot salvage, the absence of any patent vessels below the knee, reduced life expectancy (less than 6 months), immunosuppressive therapy, active neoplastic disease, or severe cognitive impairment. Also, patients who were lost to follow-up were excluded (Table 1).

### 2.1. Clinical Features

Patients with hypertension were considered those under antihypertensive therapy at the time of assessment; hypercholesterolaemia was considered in patients assuming statin therapy or in cases of impaired low-density lipoproteins (LDLs) (>55 mg/dL) needing statin therapy. Ischaemic heart disease (IHD) was considered in cases of a previous history of acute coronary syndrome or coronary revascularization, evidence of angina, or in cases of an onset of ischaemic changes in an electrocardiography test (above or under-levelling ST, q wave, inversion of T wave, and new left bundle branch block). The presence of heart failure (HF) was considered in patients with typical symptoms (i.e., dyspnoea) and echocardiographic signs of HF: reduced left ventricular ejection fraction (LVEF) (<40%) or normal or only mildly reduced LVEF and elevated levels of brain natriuretic peptides (BNPs > 35 pg/mL and/or NT-proBNP > 125 pg/mL) with a nondilated left ventricle (LV) associated with relevant structural heart disease (LV hypertrophy/left atrial enlargement) and/or diastolic dysfunction [12]. Only patients with a smoke habit at the time of assessment were considered active smokers. Cerebrovascular disease was defined in the case of documented previous cerebral ischaemia, a history of carotid artery revascularization, or in the presence of atherosclerotic plaque occluding the lumen of carotid arteries by more than 70%. Among cardiovascular risk factors, the presence of end-stage renal disease (ESRD) requiring dialysis was recorded.

### 2.2. Wound Assessment

At the first assessment, the wound characteristics (ischemia, depth, size, infection, osteomyelitis, and presence of gangrene) according to the International Working Group on the Diabetic Foot (IWGDF) definitions were recorded [13]. The diagnosis of infection was performed in relation to clinical signs (swelling, induration, redness, warmth, tenderness, purulent secretion, and pain) [14]. Bone infection (osteomyelitis) was defined as cases of bone involvement and/or exposure in association with radiological (or magnetic resonance if required) signs and the presence of an isolated strain during bone microbiological analysis [14].

The patients were treated through a local pre-set limb salvage protocol with respect to the IWGDF guidelines including antibiotic therapy (and surgery if required, offloading of the affected foot, and the close management of concomitant comorbidities) [13]. All subjects were closely managed by an MDFT, where diabetologists, including those with surgical skills, were the team leaders.

In the event of unsuccessful revascularization, the included patients received the adjuvant cell therapy using a concentrated solution of autologous PB-MNCs. PB-MNCs were administered following a local protocol known as the “*below-the-ankle approach*”, which involves an injection of MNCs into the affected foot, in accordance with the angiosome concept.

The anatomical concepts and procedures are described in detail in the following sub-sections.

### 2.3. The Wound Angiosome Concept

The angiosome theory identifies a three-dimensional area of tissue, including superficial and deeper tissue, perfused by a specific artery [15].

In the anatomical area of the foot and ankle, there are six angiosomes that originate from the three main below-the-knee arteries, namely the posterior tibial artery (PTA), the anterior tibial artery (ATA), and the peroneal artery (PA) [16].

In addition to the main angiosome vessels, there are several arterial connections between angiosomes, which often allow a rescue perfusion in the case of ischemic events that occur in a neighbouring angiosome [17]. This network is usually lacking in patients with diabetes, and this negatively affects the presence and growth of collateral vessels [17,18].

As described by Clement and Attinger, of the six angiosomes of the foot, three originate from the PTA, one from the ATA, and two from the PA [19].

The three angiosomes originating from the PTA are the following: the angiosome identified by the anatomical area of the medial malleolar surface and the medial plantar surface of the heel which is supplied by the posterior medial malleolar artery and by the branches of the posterior medial calcaneal artery; the angiosome identified by the anatomical area of the medial column and medial–plantar surface of the midfoot and forefoot, which also include the plantar surface of the hallux and the medial plantar surface of the 2nd toe, which is supplied by the medial plantar artery; the angiosome identified by the anatomical area of the lateral column and lateral–plantar surface of the midfoot and forefoot including the lateral plantar surface of the 2nd toe, the plantar surface of the 3rd and 4th toe, and the plantar and lateral surface of the 5th toe, which is supplied by the lateral plantar artery (Table 2).

The ATA provides one angiosome identified by the anatomical area of the dorsum, the dorsal surface of the toes, and the dorsal perimalleolar area, which is supplied by the dorsal pedal artery (Table 2).

The PA provides two angiosomes that can be described as follows: the angiosome identified by the anatomical area of the lateral and plantar surface of the heel, which is directly supplied by the PA and the anatomical area of the lateral surface of the rearfoot extended distally to the proximal 5th metatarsal and superiorly to the lateral malleolus, which is supplied by the lateral calcaneal branch of the PA (Table 2).

The above-mentioned description, despite potential anatomical variants, accounts for nearly 90% of cases of the anatomical characteristics of foot angiosomes [20].

Therefore, based on this angiosome anatomical description, PB-MNCs were injected along the anatomical pathway of the specific artery or arteries that supply the wound angiosome area. For instance, if the lesion was in the dorsum of the toes, PB-MNCs were injected along the dorsal pedal artery and its branches, whereas if the lesion was on the plantar surface of the 1st toe or the plantar surface of the forefoot or midfoot, PB-MNCs were injected along the medial plantar artery (Table 2).

### 2.4. Description of the Cell Therapy Procedure

The PB-MNC isolation used in the current study was produced by the point-of-care HemaTrate^®^ Blood Filtration System (Cook Regentec Indianapolis, Indianapolis, IN, USA). The cell concentration was obtained using a gravity method [21]: one hundred and twenty ml of acid–citrate–dextrose (ACD)-anticoagulated peripheral blood was loaded in the upper blood bag and gravity filtration was achieved. The concentrated MNCs were harvested through sterile saline backflush and, hence, immediately available for their administration. All procedures were performed in a dedicated foot surgery room.

**Figure 1:**
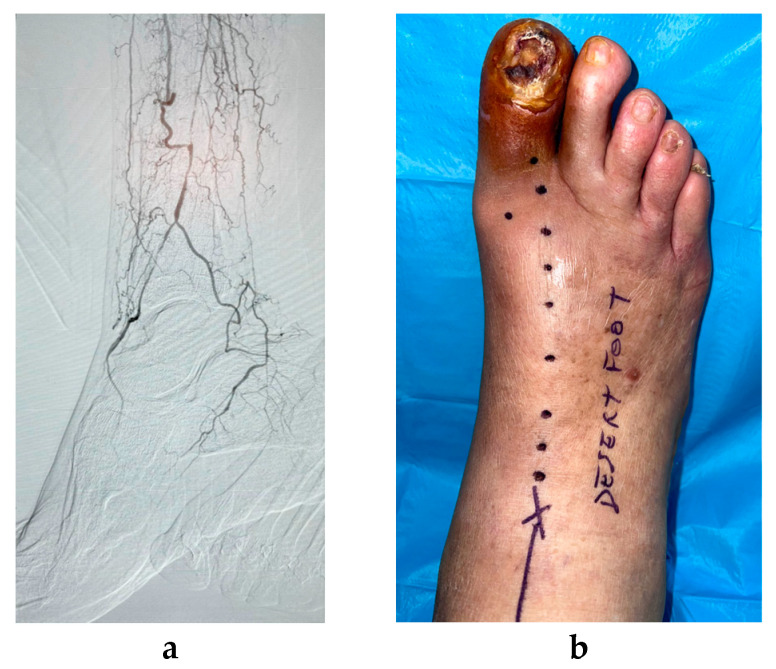
(**a**) Final angiogram picture after unsuccessful lower limb revascularization with residual “desert foot” with gangrene of the 1st toe. (**b**) Preoperative planning for the administration of PB-MNC therapy along the anatomical region of occluded pedal artery.

The PB-MNC suspension (0.1 cc in boluses) was administered via intramuscular injections into the foot along the anatomical site of the occluded wound-related artery and its collateral vessels. The technical anatomical procedure is described in detail in the section above. The bolus of PB-MNCs was injected every 1–1.5 cm and to a mean depth of 1–2 cm, using a 27-gauge needle. The first bolus was injected distally near the distal part of the patent leg artery/arteries (Figure 1).

The treatment was repeated three times every 21–42 days. From 2 to 4 weeks after the last PB-MNC treatment, all patients received surgical treatment (toe amputation, ray amputation, sequestrectomy, bone resection, transmetatarsal amputation, Lisfranc amputation, etc.) if required; otherwise, the local treatment was performed by secondary intention according to the characteristics of the primitive ulcer.

The vascular perfusion after PB-MNC and during the follow-up was evaluated through TcPO2. Even if post-treatment angiograms would be appropriate to analyse the growth and formation of new vessels in comparison to the baseline angiograms, angiographic procedures were not performed as they are not appropriate for a simple diagnostic test and so that risks related to the procedure could be avoided, such as local haemorrhage, acute renal injury due to medium-contrast injection, and hospitalization.

The study was performed according to the Declaration of Helsinki and was approved by the Ethics Committee of the University Hospital of Rome “Tor Vergata” (protocol number MC-01-2016; 151/16).

### 2.5. Outcome Measures

The primary outcome measures were healing, major amputation, and survival after 1 year of follow-up. The secondary outcome measures were an improvement in tissue perfusion evaluated through TcPO2 and foot pain defined by the numerical rating scale (NRS) recorded 2–4 weeks after the 3rd cycle of PB-MNC therapy.

### 2.6. Statistical Analysis

Statistical analysis was purely descriptive and performed by SAS (JMP12; SAS Institute, Cary, NC, USA) on a personal computer. Data are expressed as means ± SD and the continuous data for outcome are expressed as percentages.

## 3. Results

Overall, 60 patients with unsuccessful revascularization were considered for the study; 3 were excluded due to extensive tissue loss, which is not suitable for surgical foot reconstruction, and 2 were excluded for missing the complete cycle of PB-MNC therapy.

Hence, 55 patients were included. They were aged >70 years old; the majority were male and affected by type 2 diabetes with a long diabetes duration (>20 years). They reported several comorbidities, mainly IHD. Approximately 7% of them had ESRD (Table 3).

The majority of DFUs were infected and nearly 90% were assessed as gangrene. At baseline, approximately 50% of the wounds were assessed as stage 3D of the Texas University Classification (Table 3).

After PB-MNC therapy, the following surgical procedures were performed: 10 (18.2%) underwent a minor toe amputation, 2 (4.5%) underwent a first-ray amputation, 18 (32.7%) underwent a transmetatarsal amputation, 1 (1.8%) underwent a Lisfranc amputation, 4 (7.2%) underwent partial calcanectomy, and 20 (36.4%) underwent sequestrectomy/bone resection, superficial necrosectomy, or simple sharp debridement.

After 1 year of follow-up, 69.1% of patients healed and survived, 3.6% healed and deceased, 10.9% did not heal and deceased, and 16.4% had a major amputation (Table 4).

A significant increase in TcPO2 values and a concomitant reduction in pain were found comparing the baseline values recorded at the time of assessment and after the third PB-MNC injection (Table 5).

Any adverse event was recorded during the PB-MNC therapy.

## 4. Discussion

The current study showed the potential effectiveness of PB-MNC therapy as adjuvant treatment in patients with ischaemic DFUs who had unsuccessful BTA revascularization. At 1 year of follow-up, a low rate of major amputation (approximately 16%) and mortality (14.5%) were reported. The rate of major amputation was significantly lower when compared to the data we found in a previous research study on NO-CLI patients managed only by conventional therapy: 16 vs. 30%, respectively [4]. Also, the rate of mortality was significantly lower in the current study when compared to the previously cited study: 14.5 vs. 50%, respectively [4].

In addition, we found a significantly reduced rate of major amputation when the data of the current study were compared to a less recent study enrolling similar patients with BTA arterial disease and failed revascularization who were managed only by conventional therapy (16 vs. 36%) [7].

Our data are approximately similar to those found in recent analogue studies aiming to evaluate the effectiveness of PB-MNC therapy. Scatena et al. found rates of 10.5% major amputation and 20% mortality after 2 years of follow-up [22], while Panunzi et al. reported rates of 16% major amputation and 32% mortality at 1 year of follow-up [23].

In comparison to the above-mentioned studies, the results we achieved are surprisingly outstanding in relation to the characteristics of our population. Specifically, the authors also included several patients with infected DFUs and subjects with ESRD, two variables that may negatively influence outcomes, with infection and ESRD being two recognized independent predictors of amputation and mortality [2,24,25,26].

Overall, previous studies on cell therapy (both PB-MNCs or bone marrow MNCs) are not homogeneous in terms of inclusion criteria, the protocol of treatment, settings, the characteristics of PAD, and clinical outcomes. In some papers, a reduction in major amputations in NO-CLI treated by autologous cell therapy has been reported when compared to NO-CLI treated by the standard conventional therapy [9], while other studies have reported no difference in terms of limb salvage [9]. In the single clinical trial in which autologous cell therapy was compared with conservative therapy, the authors did not find a significant difference in terms of minor/major amputation at 12 weeks of follow-up (24% among patients treated with cell therapy vs. 23.5% among those treated by conservative therapy); nonetheless, subjects treated with cell therapy reported a higher rate of healing when in comparison to the conservative group (38.4 vs. 0%) [27]. However, in the cited study, the sample was very small, the follow-up was short, and the authors aimed to evaluate the impact of cell therapy on skin perfusion more than clinical outcomes.

Our current study reported a significant increase in TcPO2 values after PB-MNC therapy (from a mean of 17 mmHg at baseline to a mean of 41 mmHg 2–4 weeks after the complete cycle of cell therapy) and a significant reduction in local pain (from approximately seven points based on the numerical rating scale at the time of assessment to three points 2–4 weeks after the complete cycle of cell therapy). These data, including oxygen tension evaluation and clinical information, can easily identify an improvement in foot blood perfusion, which allows the recovery of foot ischaemia. The results we found are approximately similar to the majority of relevant studies which described a significant increase in skin perfusion and a reduction in pain after autologous cell therapy [22,23,27].

Focusing on the results we found in the current study, some points should be highlighted: (a) the authors aimed to include a cohort of patients with homogeneous vascular criteria defined by the untreatable BTA arterial disease; (b) BTA arterial disease, the most severe pattern of CLI, had the most impact on outcomes; (c) autologous cell therapy was performed via intramuscular injections of MNCs only below the ankle and along the foot “wound related artery”; (d) complex patients such as those with infected DFUs and ESRD were also included to faithfully reflect what happens in daily clinical practice.

To the best of our knowledge, this study is the first to evaluate the effectiveness of PB-MNC therapy in very complex patients with specifically unsuccessful BTA revascularization and extensive tissue loss.

In addition to the severe vascular pattern, a large number of patients reported forefoot, midfoot, or heel gangrene, requiring a surgical procedure such as transmetatarsal/Lisfranc amputation or partial calcanectomy in approximately 40% of cases.

Based on clinical, vascular, and foot characteristics, the achieved results are highly promising for improving healing, limb salvage, and survival by using PB-MNC therapy in patients with NO-CLI.

The effectiveness of autologous cell therapy in patients with desert foot or severe small artery disease such as those enrolled in this study may be explained by its mechanism of action, which promotes the development of collateral vessel and vessel remodelling in the wound angiosome area [23].

The growth of new collateral vessel seems related to the ability of PB-MNCs to induce vascular remodelling through paracrine activity involving different actors, such as growth factors, cytokines, angiogenic coding and noncoding RNAs (either free or encapsulated in extracellular vesicles), and small-size exosomes [28,29,30]. Hence, when the direct wound-related artery is occluded, the presence of new collateral vessels can supply the specific angiosome area.

In addition, MNCs can induce the polarization of macrophage M1 (an inflammatory phenotype) to the macrophage M2 (a regenerative phenotype), promoting the healing pathway of chronic ulcers, including ischaemic ulcers [31,32,33].

The study had some limitations. It was a monocentric study, and the sample size was not so large; nonetheless, it seems acceptable in comparison to the sample size described in similar studies. The authors fully understand that the major issue of the study was the lack of a control group. Even though the data achieved may be considered outstanding, the absence of a control group significantly reduces the strength of the study.

Further studies are mandatory to better define indications (both clinical and vascular), the timing of treatment, repeatability, etc.

In particular, randomized controlled trials (RCTs) are required to support the robustness of this kind of study and potential indications for PB-MNCs. In our personal view, an RCT should include patients with NO-CLI who have had unsuccessful revascularization below the ankle (desert foot and/or an absence of direct flow to the wound angiosome area with TcPO2 < 30 mmHg), with a randomized identification of two groups with the same clinical and wound characteristics to avoid any bias of enrolment: a study group managed by active therapy (cell therapy plus the best standard of care) and a control group managed by conservative therapy (placebo + the best standard of care). The outcome measures should be evaluated at 6–12 months of follow-up and may be like those considered in the current study, including healing, major amputation, survival, an improvement in foot perfusion, and pain.

## 5. Conclusions

This study confirms that PB-MNC therapy can be a rescue therapy in critical patients with ischaemic DFUs and NO-CLI, which are often considered as “unsalvageable feet”. Accordingly, the authors reaffirm that, in the near future, those patients considered until now as “hopeless subjects” may also have a new chance to save their feet and improve their life expectancy. This frontier has now been opened by autologous cell therapy, which is offering new solutions for the management of severe foot ischaemia not treatable by ordinary revascularization.

## Figures and Tables

**Table 1:** Inclusion and exclusion criteria for the study enrolment.

**Inclusion Criteria**
- Ischaemic/neuro-ischaemic DFU stage 1C-D, 2C-D, 3C-D according to Texas University Classification; - Unsuccessful revascularization below the ankle: desert foot and/or an absence of direct flow to the wound angiosome area with TcPO2 < 30 mmHg; - Patency of at least one vessel below the knee (anterior tibial artery, posterior tibial artery, and peroneal artery).
**Exclusion criteria**
- Indication for primary amputation due to an absence of anatomical suitability for surgical foot salvage; - An absence of any vessels below the knee; - Reduced life expectancy (less than 6 months); - Immunosuppressive therapy; - Active neoplastic disease; - Severe cognitive impairment; - Lost to follow-up.

At the baseline assessment, demographics, clinical and wound characteristics were recorded.

**Table 2:** Description of foot angiosomes and targeted arteries for cell injection according to the wound location.

Foot Angiosome and Anatomical Vascular Site of Cell Injection	Wound Location
**Posterior tibial artery**	The medial–plantar surface of the heel; The medial column and medial–plantar surface of the midfoot and forefoot, the plantar surface of the hallux, and the medial–plantar surface of the 2nd toe; The lateral column and lateral–plantar surface of the midfoot and forefoot, the lateral plantar surface of the 2nd toe, the plantar surface of the 3rd and 4th toe, and the plantar and lateral surface of the 5th toe.
(1) The posterior medial malleolar artery and posterior medial calcaneal artery; (2) The medial plantar artery; (3) The lateral plantar artery.	
**Anterior tibial artery**	The dorsum of the foot, the dorsal surface of the toes, and the dorsal perimalleolar area.
(1) The pedal artery and its branches (arcuate artery and intermetatarsal arteries).	
**Peroneal artery**	The lateral and plantar surface of the heel; The lateral surface of the rearfoot extended until the proximal 5th metatarsal and superiorly until the lateral malleolus.
(1) The distal part (below the ankle) of the peroneal artery; (2) The lateral calcaneal branch of the peroneal artery.	

**Table 3:** Baseline characteristics at the assessment of the whole population. HbA1c: glycated haemoglobin. ESRD: end-stage renal disease. IHD: ischaemic heart disease. Hb: haemoglobin.

Variable	Value
Age (years)	74.8 ± 5.8
Sex (male) n (%)	39/55 (70.9)
Diabetes (type 2) n (%)	51/55 (92.7)
Diabetes duration (years)	22.1 ± 7.7
HbA1c mmol/mol (%)	58 ± 9 (7.4 ± 3)
Dyslipidaemia n (%)	29 (52.7)
Hypertension n (%)	53 (96.3)
ESRD n (%)	4 (7.3)
IHD n (%)	47 (85.4)
Heart failure n (%)	5 (9.1)
CVD n (%)	21 (38.2)
Smoke	4 (7.3)
Hb (gr/dL)	12.1 ± 1.2
Ulcer size (>5 cm^2^) n (%)	41 (74.5)
Depth (bone involvement)	36 (65.4%)
Infection n (%)	45 (81.8)
Osteomyelitis n (%)	28 (50.9)
Gangrene n (%)	49 (89.1)
Heel location n (%)	13 (23.6)
Texas University Classification	
1C n (%) 2C n (%) 3C n (%) 1D n (%) 2D n (%) 3D n (%)	2 (3.6%) 1 (1.8%) 8 (14.5%) 1 (1.8%) 15 (27.3) 28 (50.9)

**Table 4:** Primary outcomes of the whole population.

Primary Outcomes	Value
Healed and survived n (%)	38 (69.1)
Healed and deceased n (%)	2 (3.6)
Not healed and deceased n (%)	6 (10.9)
Not healed and amputated n (%)	9 (16.4)

**Table 5:** Secondary outcomes of the whole population. TcPO2: Transcutaneous Oxygen Pressure. NRS: numerical rating scale.

Secondary Outcomes	Before PB-MNC	After PB-MNC	*p*-Value
TcPO2 (mmHg)	17 ± 11	41 ± 12	<0.0001
Pain (NRS)	6.8 ± 1.7	2.8 ± 1.7	<0.0001

## Data Availability

Data were recorded by authors in a local database. Data were not available for privacy and ethical restrictions.
